# Malignant giant cell tumor of toe: A case report and review of literature

**DOI:** 10.1097/MD.0000000000029471

**Published:** 2022-06-03

**Authors:** Kazuhiko Hashimoto, Shunji Nishimura, Tomohiko Ito, Masao Akagi

**Affiliations:** Department of Orthopedic Surgery, Kindai University Hospital, Osaka-Sayama City, Japan.

**Keywords:** case report, giant cell tumors, malignant, toes

## Abstract

**Introduction:**

A giant cell tumor of soft tissue (GCST) is a benign soft tissue tumor that often occurs subcutaneously in the extremities. Rare cases of malignant GCST have been reported, but its pathogenesis remains unclear.

**Patients concerns:**

We report a case of a 68-year-old man who noticed a painless mass on his second toe one and a half years ago. He visited the Department of Dermatology at our hospital. Magnetic resonance imaging revealed a soft tissue tumor, surrounding the distal aspect of the second toe.

**Diagnosis:**

A biopsy of the tumor was performed by a dermatologist, and it revealed a malignant giant cell tumor of the toe.

**Interventions:**

He was referred to our department and underwent lay amputation for wide-margin resection.

**Outcomes:**

No recurrence or metastasis was observed 5 years after treatment.

**Conclusion:**

: Malignant GCST should be treated with wide-margin resection immediately after its diagnosis.

## 1 Introduction

Giant cell tumor of soft tissue (GCST) was first reported in 1972 by Salm and Sissons.^[1]^ GCST is morphologically similar but genetically unrelated to giant cell tumors of the bone.^[2]^ GCST predominantly affects patients in the fifth decade of their life, but it affects patients aged 5–89 years old as well.^[2]^ There is no apparent difference in the incidence according to sex or ethnicity.^[2]^ GCST is typically benign and painless. However, some tumors have exhibited aggressive clinical features.^[3]^ Previous reports have documented the low malignant potential of GCST.^[4,5]^ However, clinical and pathologic findings, suggestive of a malignant GCST, have not been investigated.^[2]^ Herein, we describe a case of GCST of the toe that presented with malignant features.

## 2 Case report

The patient was a 68-year-old man, who noted a painless mass on the second toe of his right foot one and a half years ago. The mass reportedly enlarged, and the patient consulted at our hospital.

**Figure 1:**
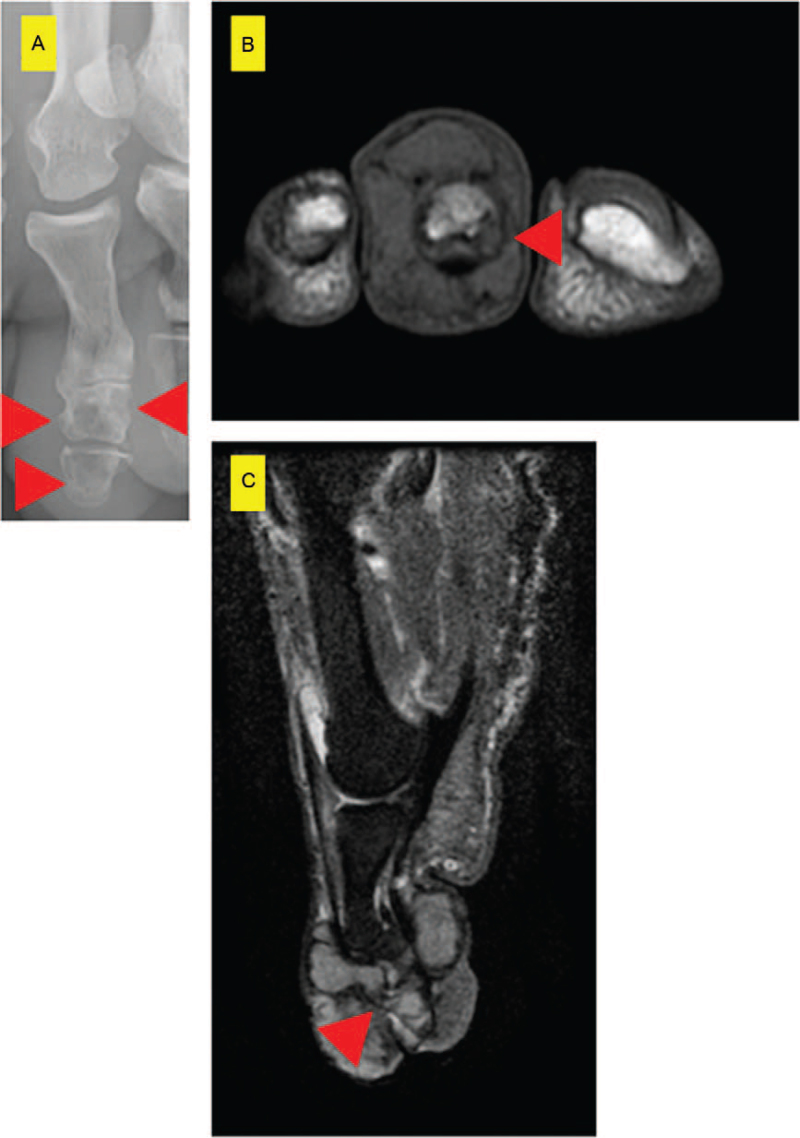
(A) The radiograph showed a soft tissue shadow enhancement at the distal end of the second toe and lytic lesions (red arrowheads) in the intermediate and distal phalanges. (B) Coronal-view magnetic resonance imaging (MRI) revealed a low-intensity mass surrounding the second toe. Partial invasion of the tumor into the bone was observed. Red arrowheads indicate lytic lesions in the bones. (C) Sagittal-view MRI revealed a high-intensity mass surrounding the second toe. Partial invasion of the tumor into the bone was observed. Red arrowheads indicate lytic lesions in the bones.

**Figure 2:**
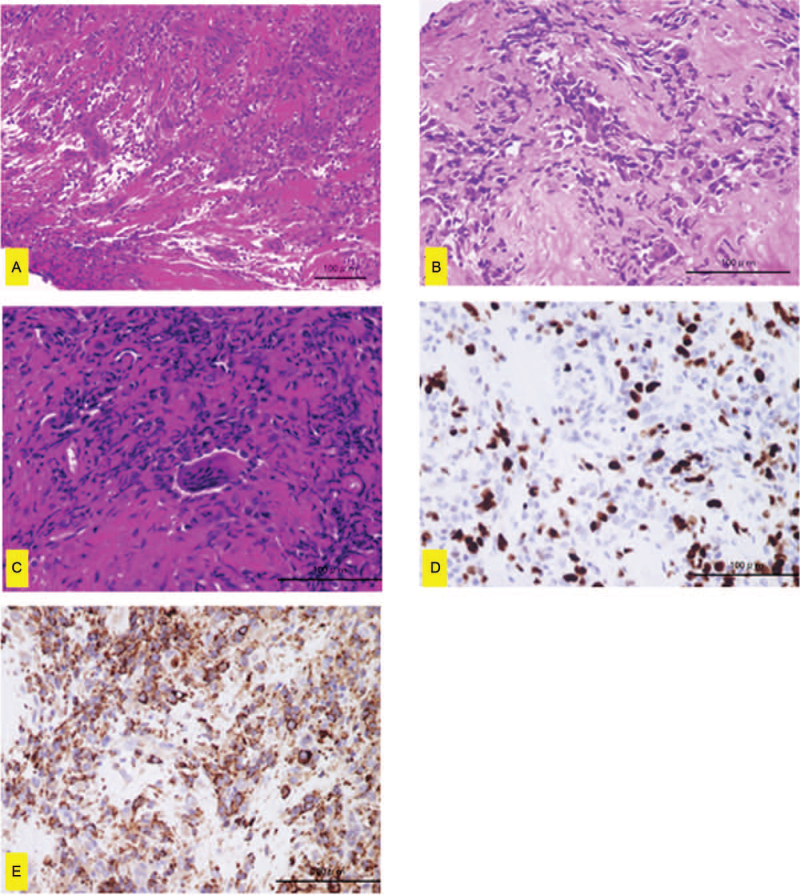
(A) Histopathological findings of the biopsy in hematoxylin-eosin (H&E) staining in lower magnification (×200). (B) Histopathological findings of the biopsy in H&E staining in higher magnification (×400). (C) Histopathological findings of the specimen harvested during amputation in H&E staining in lower magnification (×400). (D) Histopathology revealed diffuse proliferation of atypical cells with enlarged round nuclei. Some multinucleated giant cells were also present. The Ki-67 positive cell rate was approximately 30%. (E) Immunohistochemical staining for CD-68 was observed. The scale bars = 100 μm.

**Figure 3:**
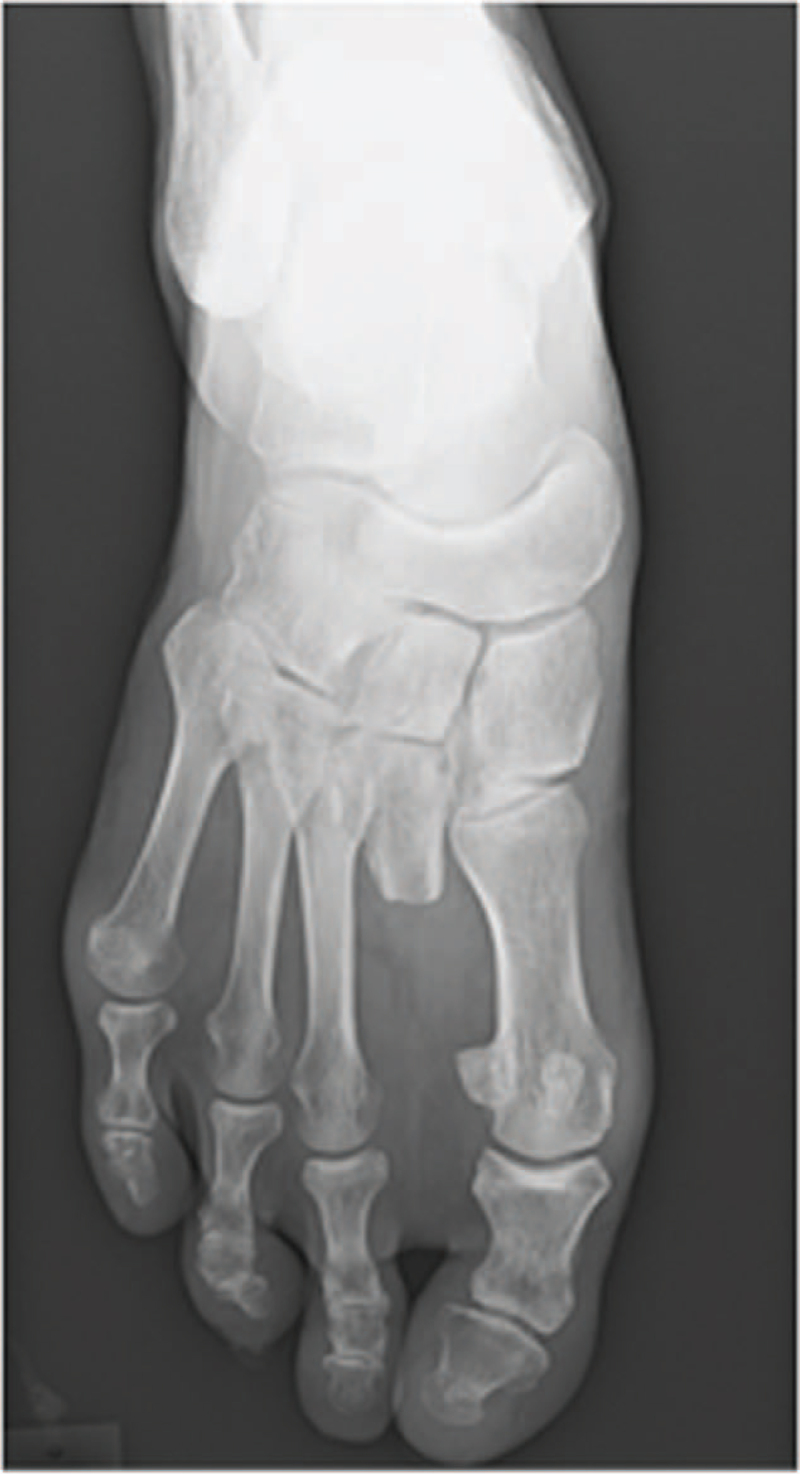
The radiograph of the right foot after the surgical treatment.

Clinical examination revealed a nodular mass, measuring 40 × 30 × 22 mm, on the second toe of the patient's right foot, along with a skin ulcer. There was also a 21-mm granulomatous nodule on the nail bed with clear borders. No tenderness was observed. The radiograph showed a soft tissue shadow enhancement at the distal end of the second toe and lytic lesions in the intermediate and distal phalanges (Fig. 1A). Magnetic resonance imaging (MRI) showed a low-intensity mass, surrounding the second toe, on T1-weighted imaging and a high-intensity mass on T2-weighted imaging (Fig. 1B and 1C). Histopathology revealed diffuse proliferation of atypical cells with enlarged round nuclei (Fig. 2A and 2B). Some multinucleated giant cells were also present (Fig. 2A and 2B). Five mitotic cells per 10 high-power fields (HPF) were observed (Fig. 2C). The Ki-67 positive cell rate was approximately 30% (Fig. 2D). Immunohistochemical staining for CD-68 was observed (Fig. 2E). The patient was diagnosed with malignant GCST. Based on the histologic findings of a malignancy, lay amputation was performed from the midshaft of the second metatarsal bone (Fig. 3). Five years after postoperatively, neither recurrence nor metastasis was observed.

## 3 Discussion

GCST is morphologically similar but genetically unrelated to giant cell tumors of the bone. The World Health Organization classifies this type of tumor into low-grade and high-grade GCSTs (2). The etiology of GCST has not been identified. GCST typically occurs in the superficial soft tissues of the extremities (70% of tumors). Other sites include the trunk (20%) and head and neck (7%) (2). GCST mostly develops in the lower extremities (50%),^[3]^ and almost all cases exhibit benign clinical and histological features.^[3]^ To the best of our knowledge, this is the first report of GCST of the toe presenting with malignant histological findings.

GCSTs are painless masses with an average duration of 6 months (2). Malignant tumors grow rapidly.^[6]^ Moreover, the skin ulcers of a soft tissue sarcoma signify a malignancy.^[7,8]^ In the present case, a painless and rapidly growing mass with skin ulcers was observed.

A GCST should be differentiated from other benign soft tissue tumors, such as lipomas and schwannomas.^[8,9]^ The radiographic features of GCST include increased soft tissue shadows and bony changes.^[10–12]^ An increased soft tissue shadow was observed in more than half of cases,^[10–12]^ while calcification was reported in 5.6% to 16.7% of cases.^[11,12]^ In addition, bone erosion is a common radiographic finding, found in 6.2% to 33.3% of GCST cases.^[10–12]^ MRI provides high-resolution images and is useful for diagnosing soft tissue tumors.^[13]^ GCST typically appears as a solid, homogeneous, hypoechoic mass on MRI.^[10]^ However, Hu et al showed that heterogeneous T1- and T2-weighted images of a mediastinal GCST suggested malignancy.^[9]^ According to recent literature, malignant GCST exhibits a moderate degree of mosaicism on T1-weighted imaging and high-intensity mosaicism on T2-weighted imaging.^[14]^ In the present case, bone erosion was observed on radiography, but calcification was not noted. However, there were no mosaic findings on MRI.

Histologically, GCST is characterized by its multinodular architecture.^[2]^ Cellular nodules are composed of a mixture of round cells and multinucleated giant cells.^[2]^ Benign GCST consists of mononuclear cells, lacking nuclear atypia or pleomorphism, and the mitotic rate within this population is low (average, 3 mitotic cells per 10 HPF).^[3]^ In contrast, malignant GCST consists of mononuclear cells, exhibiting anisocytosis, nuclear atypia, pleomorphism, high mitotic activity that includes atypical forms (mean, 25 mitoses per 10 HPF), and necrosis.^[3,15]^ On immunohistochemical examination, GCST was reportedly positive for vimentin, CD163, CD68, and CD34 (vascular). Meanwhile, it was negative for SMA, CK, S-100, and desmin.^[3,9,15]^ However, the diagnostic immunostaining pattern has not been determined.^[2]^ Ki-67 is used to evaluate tumor cell proliferation.^[16]^ The mean Ki-67 percentage of soft tissue sarcoma is 20%–25%.^[17,18]^ Based on previous reports, the Ki-67 positivity rate of malignant GCST reached up to 20-30%.^[9,19]^ In the current case, the tumor was positive for CD68, and this finding was consistent with the previous reports. Although the mitotic cell count was not very high, the Ki-67 positive rate and malignant GCST were high.

Surgical resection is the main treatment option for GCST.^[11]^ Wide resection margins are necessary for the removal of malignant soft tissue tumors to obtain favorable outcomes.^[20]^ The main treatment for GCST is surgical resection with or without radiotherapy and chemotherapy. Patients undergoing surgery only had a more favorable prognosis than those undergoing surgery and radiotherapy.^[21]^ The standard resection method for GCST has not been determined because the pathogenesis of the tumor remains unclear.^[9]^ In the present case, a wide resection margin was necessary because the histological findings suggested a malignancy. Therefore, lay amputation was performed to obtain wide resection margins.

GCST recurrence is rare with a local recurrence rate of 12%.^[2]^

Several studies on the prognosis of malignant giant cell bone tumors have been published, and the 5-year survival rate varied from 50% to 87%.^[22,23]^ Although no recurrence or metastasis has been observed in the present case, careful follow-up is needed.

## 4 Conclusion

We reported the treatment of a rare case of malignant GCST, originating in the toes. GCST with malignant histopathological findings should be treated with wide-margin resection immediately after diagnosis.

## Acknowledgments

The authors would like to thank Editage (www.editage.jp) for English language editing.

## Author contributions

**Conceptualization:** Kazuhiko Hashimoto, Masao Akagi.

**Data curation:** Kazuhiko Hashimoto, Shunji Nishimura, Data curation.

**Formal analysis:** Kazuhiko Hashimoto, Masao Akagi, Shunji Nishimura.

**Investigation:** Kazuhiko Hashimoto, Masao Akagi, Shunji Nishimura, Data curation, Investigation.

**Methodology:** Kazuhiko Hashimoto, Shunji Nishimura, Investigation.

**Project administration:** Kazuhiko Hashimoto.

**Resources:** Kazuhiko Hashimoto, Shunji Nishimura, Investigation.

**Software:** Kazuhiko Hashimoto.

**Supervision:** Masao Akagi, Shunji Nishimura.

**Validation:** Kazuhiko Hashimoto, Masao Akagi, Shunji Nishimura.

**Visualization:** Kazuhiko Hashimoto.

**Writing – original draft:** Kazuhiko Hashimoto, Masao Akagi, Shunji Nishimura, Investigation.

**Writing – review & editing:** Kazuhiko Hashimoto, Masao Akagi, Shunji Nishimura, Investigation.
